# The Effect of Variable-Pitch Headless Compression Screws and Cortical Screws on Interfragmentary Compression: An In Vitro Polyurethane Foam Block Model

**DOI:** 10.3390/ani16071126

**Published:** 2026-04-07

**Authors:** Brendan R. Castellino, Daniel J. Wills, Christopher J. Tan, Max J. Lloyd, William R. Walsh

**Affiliations:** 1 Sydney School of Veterinary Science, University of Sydney, Camperdown 2050, Australia; ctan@sashvets.com; 2 Surgical and Orthopaedic Research Laboratories (SORL), School of Clinical Medicine, UNSW Sydney, Sydney 2033, Australia; d.wills@unsw.edu.au (D.J.W.); max.lloyd@unsw.edu.au (M.J.L.); w.walsh@unsw.edu.au (W.R.W.); 3 Small Animal Specialist Hospital (SASH), Sydney 2113, Australia

**Keywords:** headless compression screws, cortical lag screws, interfragmentary compression, fracture repair, area of compression

## Abstract

Articular fractures require precise anatomical reduction and rigid fixation to heal appropriately. In veterinary cases that involve fracturing of the lateral humeral condyle, cortical bone screws inserted in lag fashion with Kirschner wire are the preferred method for surgical fixation. However, relatively high complication rates associated with cortical lag screws (CLSs) highlights the need to investigate alternate screw designs. Variable-pitch headless compression screws (VPHCSs) are unique as they advance beneath the cortical surface. Although the use of VPHCSs are widely utilised in human orthopaedics, the current use in veterinary orthopaedics is limited. This study aimed to evaluate the peak interfragmentary force (PIF) and area of compression (AOC) generated by a 3.5 mm self-tapping cortical screw placed in lag fashion and a 3.5 mm VPHCS inserted to four depths. PIF and AOC were measured using a pressure-sensitive film placed between two blocks of polyurethane foam (0.24 g/cm^3^), simulating a transverse fracture. CLSs were inserted by hand into predrilled 2.5 mm pilot holes. PIF and AOC were measured at full insertion. VPHCSs were placed into predrilled 2.5 mm pilot holes, followed by a 3.5 mm tapered countersink. The screw was inserted until the head was level with the surface. PIF and AOC were measured between the two blocks. The screw was continued until the head was at a depth of 2, 5, and 9 mm below the surface, and the PIF and AOC were measured again at each stage. There was no detectable difference in PIF and AOC between CLSs and VPHCSs countersunk to −2 mm (PIF–CLS: Mean = 12.886, SD = 2.370; 2 mm: Mean = 17.301, SD = 8.858, *p* = 0.319; AOC–CLS: Mean = 0.936, SD = 0.291; 2 mm: Mean = 0.925, SD = 0.447, *p* = 0.872). VPHCSs countersunk to −5 mm and −9 mm produced significantly greater PIF compared to CLSs (5 mm: Mean = 16.086, SD = 6.799, *p* = 0.002; 9 mm: Mean = 34.987, SD = 4.015, *p* < 0.001). VPHCSs countersunk to −5 and −9 mm produced significantly greater PIF and AOC compared to CLSs in this model. Further investigation is required to produce recommendations for clinical use.

## Simple Summary

Correct anatomical bone healing is crucial in joint fracture repair, especially in canine cases involving the lateral portion of the humeral condyle. Although cortical bone screws are often used to repair these fractures, high complication rates have been reported. Consequently, attention has shifted toward alternative implant designs, including variable-pitch headless compression screws. These screws are uniquely designed to be buried beneath the bone surface, thereby eliminating complications associated with cortical screw-head prominence. However, the current use of variable-pitch headless compression screws in veterinary orthopaedics is limited. In this study, we used a polyurethane foam block fracture model with pressure-sensitive film to investigate how variable-pitch headless compression screws inserted to varying depths compared to standard cortical bone screws. We found that variable-pitch headless compression screws, when inserted 5 mm and 9 mm below the foam surface, produced greater magnitudes of interfragmentary compression than cortical bone screws. Additionally, variable-pitch headless compression screws, when inserted 2 mm below the foam surface, produced fracture compression comparable to that of cortical bone screws. We recommend further in vivo testing to support the clinical application of variable-pitch headless compression screws in articular fracture repairs.

## 1. Introduction

Screws are simple machines that convert torque into linear motion, pushing objects along the screw’s axis [1,2]. As a result, they are a fundamental component of internal fixation, used in both human and veterinary surgical worlds, including spine, orthopaedics, and craniomaxillofacial disciplines [1,3,4,5]. In veterinary orthopaedics, interfragmentary screws are frequently used in articular fractures involving the lateral portion of the humeral condyle, which account for 56–67% of all canine humeral condyle fractures globally [6].

Historically, precise reduction, rigid internal fixation, preserved blood supply, and early mobilisation have been advocated to achieve successful healing of articular fractures [7]. Using anatomically correct reduction combined with adequate compressive force generates rigid fixation and subsequent interfragmentary compression (IFC) [5]. IFC increases fixation stability by preventing shear forces and micromotions along the fractured surface, promoting primary bone healing [8]. Primary bone healing is desirable in articular fracture repair, as secondary bone healing relies on callus formation, which may lead to joint incongruity and reduced range of motion [5,9]. Recently, IFC has received renewed interest in the veterinary biomechanical testing literature, particularly in the performance of various fixation constructs, including cortical bone screws and Tibial Plateau Levelling Osteotomies (TPLOs) [5,10].

Various fixation options are available to the surgeon to produce IFC, including cortical bone screws, self-compressive Orthofix pins, Kirschner wire (K-wire), compression plates, and headless compression screws [5,11,12,13,14,15,16]. Among these, screw-based fixation has been identified as a key proponent of IFC generation, particularly with respect to certain screw geometric characteristics. These include thread pitch, thread diameter, core diameter, thread depth below the cortex, screw length, head design, and overall screw diameter, all of which have been shown to correlate with IFC [17,18,19,20,21,22,23].

Cortical bone screws can be inserted in lag fashion, i.e., passing through a glide hole drilled in the near fragment and engaging a pilot hole in the far fragment [2]. This relies on the head of the screw to abut against the cortical surface, pulling the two bone fragments together, generating IFC [2]. Although cortical lag screws (CLSs) are commonly used in the fixation of fractures involving the lateral portion of the humeral condyle, relatively high major complication rates have been reported, ranging between 17.5% and 28% [14,24,25]. These complications include seroma formation, due to soft tissue irritation, caused by either the screw head or K-wire abutment, surgical site infection, and transcondylar screw migration or failure [16,26]. Contact between the screw head and the cortical bone may lead to resorption and erosion of the underlying cortical surface due to micromotion or pressure necrosis [27]. Additionally, further axial traction is prevented as the head contacts the surface of the bone. Consequently, continued rotation of the screw results in stripping of the screw–bone interface, which could lead to screw loosening and result in implant failure [27,28].

Variable-pitch headless compression screws (VPHCSs) offer a unique advantage as, without a head, they continue to advance past the cortical surface with continued rotation, whilst continuing to generate IFC [29]. This allows them to be buried beneath the cortical surface, eliminating screw-head abutment and soft tissue impingement. VPHCSs are considered second-generation designs and represent an advancement by generating greater compression over first-generation headless compression screws, such as the Herbert screw [29]. Additionally, second-generation headless compression screws are cannulated, allowing percutaneous fixation over a guidewire, reducing overlying tissue disruption and preserving the blood supply [17,29,30,31]. In human scaphoid fracture studies, Acutrak screws, a second-generation VPHCS, have been shown to generate superior fixation compared to the traditional Herbert screws [32,33]. Furthermore, Acutrak screws have demonstrated a higher torque threshold before loss of fixation and greater resistance to cyclical bending and loading compared to Herbert screws in foam scaphoid models [34,35]. Although VPHCSs are frequently studied in equine metacarpal, human scaphoid, and lateral malleolar models, a direct comparison of these results to the clinical canine scenario is limited due to differences in skeletal anatomy, bone quality, and density [31,33,36,37,38,39]. Additionally, while the literature recommends a 1–2 mm countersink depth, optimal depth may vary between headless compression screw types in order to achieve effective compression [17].

This study aimed to evaluate the mechanical properties of the cortical bone screws and VPHCSs, specifically the peak interfragmentary force (PIF) and area of compression (AOC), to identify characteristics that might influence the clinical use of the VPHCS in the clinical scenario of canine humeral condylar fracture repairs. The null hypothesis was that there would be no difference in PIF and AOC generated by cortical bone screws compared to VPHCSs, irrespective of countersinking depth. Cortical bone screws were placed in lag fashion (CLS), whilst VPHCSs were placed to four depths relative to the bone surface, i.e., 0, 2, 5, and 9 mm.

## 2. Materials and Methods

### 2.1. Fracture Model

Closed-cell rigid polyurethane (PU) foam (Sawbones^®^, Washington, DC, USA) blocks were used as a surrogate model for the lateral humeral condyle (LHC) fracture. A PU foam density of 0.24 g/cm^3^ (PCF15) with a compressive strength of 2–4 MPa was chosen to best represent canine humeral trabecular bone [39,40]. A simple, transverse fracture was simulated using two foam blocks measuring (132 mm × 182 mm × 20 mm).

Fractures were reduced and fixed with either cannulated, self-tapping, fully threaded VPHCSs (3.5 mm; VPC340, Knight Benedikt^®^, Seven Hills, NSW, Australia) or non-cannulated, self-tapping, cortical bone screws (3.5 mm; KOE400S, Knight Benedikt^®^, Seven Hills, NSW, Australia) as CLSs.

A priori power analysis using G*Power 3 was conducted to determine the appropriate sample size for the main study [41]. A pilot study consisting of three groups (*n* = 8 per group) was used to estimate the effect size. Using an α of 0.05, a desired statistical power of 0.80, and an estimated effect size of 0.67 (test statistic = 15.387), the analysis indicated that a minimum of 3 samples per group was required. To ensure sufficient statistical power, the final study used 10 samples per group.

### 2.2. Screw Analysis

**Figure 1:**
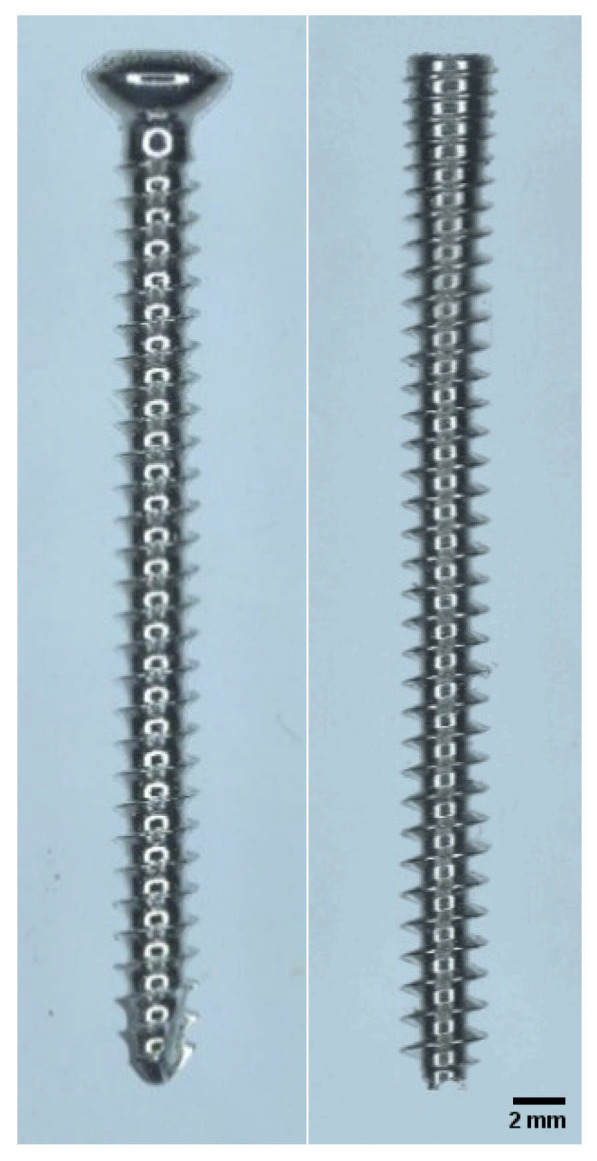
Stereozoom images of a 3.5 mm self-tapping cortical bone screw (**left**) and 3.5 mm fully threaded Variable-pitch headless compression screw (**right**), both 40 mm in length.

Screw types were imaged using stereozoom microscopy (LED5000 CXI, Leica Microsystems, Wetzlar, Germany). Measurements of screw length, head diameter, core diameter, thread diameter, thread pitch, and thread height were made in the imaging software (Figure 1 and Table 1).

### 2.3. Interfragmentary Compression and Area of Compression

**Figure 2:**
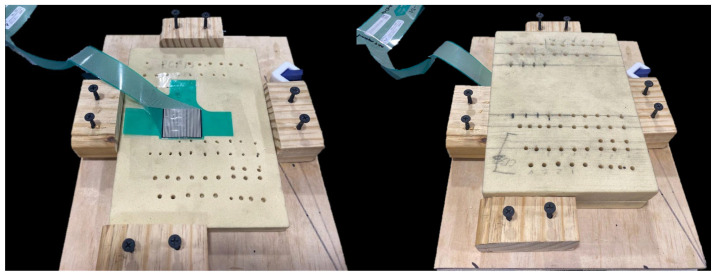
(**Left**): Distal foam block secured in a custom wooden jig with pressure-sensitive film positioned adjacent to the pilot hole. The proximal foam block was removed for illustrative purposes to demonstrate pressure-sensitive film placement. Prior to film placement, a 2.0 mm pilot hole was drilled with the proximal block positioned on top of the distal block (hole visible adjacent to the centre of the film). (**Right**): Final configuration showing the proximal foam block positioned on the distal block with the pressure-sensitive film between them. The location of the film was varied to ensure adjacent placement to the hole for each screw test.

A calibrated pressure-mapping sensor film (Model 4000, Tekscan Inc, Boston, MA, USA) was placed between the two foam blocks, immediately adjacent to the predrilled hole, before implant fixation (Figure 2). A wooden board with holding blocks acted as a jig to allow the reproduction of block positioning and prevent the sliding of the blocks during drilling and screw insertion.

### 2.4. Insertion Technique

CLS placement was performed according to the standard AO technique [21]. For the CLS fixation, a pilot hole was created with a 2.5 mm drill bit (JBE250, Knight Benedikt^®^, Seven Hills, NSW, Australia) through both blocks. A 3.5 mm drill bit (JBE350, Knight Benedikt^®^, Seven Hills, NSW, Australia) was then used to enlarge the pilot hole in the proximal foam block. After sensor film placement adjacent to the distal block’s pilot hole, the upper block was placed on top of the film without additional force, and the sensor load was zeroed. A 3.5 mm CLS was placed in the predrilled hole and tightened with a screwdriver attached to a load cell to measure insertion torque (SWS-250, Transducer Techniques LLC, Temecula, CA, USA). During pilot testing, the head of the CLS was observed to compress the foam after contact with the head or strip the threads above a torque measurement of 0.25 Nm; therefore, screws were inserted to a maximum torque of 0.2 Nm. PIF and AOC were measured for 5 s post-insertion to remove operator-induced axial forces and allow foam adaptation. The protocol was repeated ten times in the same foam blocks by the same operator (BC), with each screw spaced 10 mm apart between insertions to ensure independence between tests.

**Figure 3:**
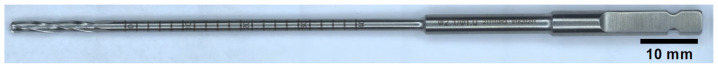
Stereozoom image of a 2.0 mm cannulated drill bit used to drill pilot holes in the proximal and distal foam blocks before the pilot hole is enlarged with a 3.5 mm cannulated countersink drill bit.

**Figure 4:**
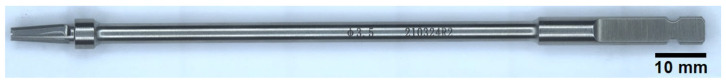
Stereozoom image of a 3.5 mm cannulated countersink drill bit used to enlarge pilot holes in the proximal foam block before insertion of a VPHCS. The drill bit had a conical taper, with a diameter reducing from 3.5 mm to 2.2 mm across a 10 mm segment.

For the VPHCS fixation, the manufacturer’s instructions were used to guide insertion. A 2.0 mm cannulated drill bit (VPC387, Knight Benedikt^®^, Seven Hills, NSW, Australia) was used to create a pilot hole through both foam blocks (Figure 3). A cannulated 3.5 mm countersink drill bit (VPC386, Knight Benedikt^®^, Seven Hills, NSW, Australia) was then used to enlarge the pilot hole in the proximal foam block (Figure 4). After sensor film placement adjacent to the distal block’s glide hole, the upper block was placed, and the sensor zeroed, before screw insertion. Initially, VPHCSs were inserted flush with the surface (0 mm). Callipers were then used to measure the countersinking distance to −2 mm, −5 mm, and −9 mm below the upper foam block surface. PIF and AOC were measured 5 s post-insertion to minimise operator-induced axial forces and allow foam adaptation. As with the CLS fixation, 10 samples were run with screws placed 10 mm apart by the same operator (BC).

### 2.5. Statistical Analysis

Five groups were evaluated, each with 10 samples, i.e., cortical bone screw, and VPHCSs at 0 mm depth, 2 mm, 5, and 9 mm. Data analysis was performed using IBM SPSS Statistics Version 30.0. Normality was assessed qualitatively with Q-Q plots, boxplots, and histograms and quantitatively with Shapiro–Wilk tests—normality of data was assumed if *p*-value > 0.05. Since the data was not normally distributed, Kruskal–Wallis ANOVA and Dunn’s post hoc analysis with Bonferroni correction were used to detect significant differences between groups. Additionally, simple linear regression was used to test if the VPHCS countersink depth significantly predicted PIF and AOC. A *p*-value < 0.05 was considered significant.

**Table 1:** Measurements of variable pitch headless compression screw (VPHCS) and cortical bone screw inserted in lag fashion (CLS) using imaging software. Screw measurements were taken from the screw head to the screw point. Measurement with ranges, i.e., thread diameter 3.85 mm–3.5 mm, indicates the full, variable linear length of the screw from head to point. (*) Measurements from the screw tip also delineate the cutting flute section, accounting for the identical thread pitch observed at the head and tip. Figure 5 illustrates how core diameter, thread diameter, thread pitch, and thread height change along the VPHCS length.

	VPHCS	CLS
Length	40 mm	40 mm
Head diameter	3.5 mm	6.0 mm
Core diameter	3.5 mm–1.85 mm *	2.4 mm
Thread diameter	3.85 mm–2.35 mm *	3.5 mm
Thread pitch	0.80 mm–0.80 mm *	1.25 mm
Thread height	0.35 mm–0.50 mm *	0.55 mm

**Figure 5:**
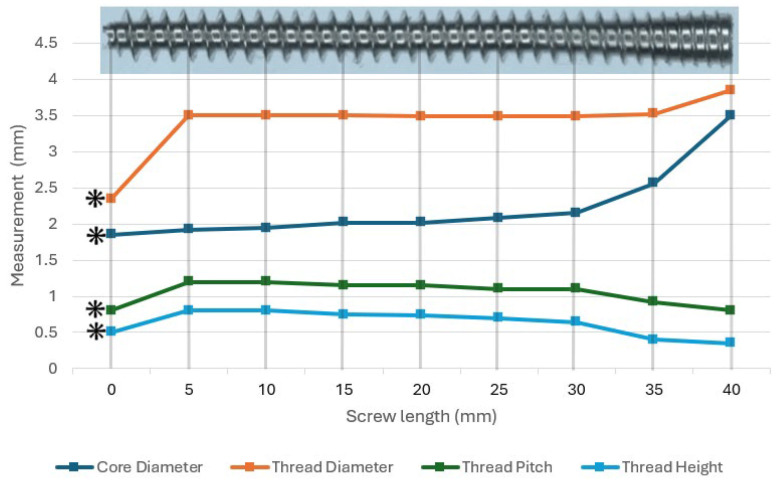
Line graph depicting the variation in core diameter, thread diameter, thread pitch, and thread height from the head (0 mm) to the point (40 mm) of a VPHCS. Faint vertical gridlines visually align the plotted data with a 40 mm reference image of the screw. Asterisks (**⁕**) indicate dimensions measured specifically at the cutting flute section.

## 3. Results

### 3.1. Screw Characterisation

After the initial cutting flute, the core diameter, thread diameter, thread pitch, and thread height remain constant over approximately 75% of the screw length. In the final 15% of the VPHCS head region, thread pitch decreases by 27% and thread height by 45%. Over the same region, thread diameter and core diameter increase by 10% and 63%, respectively (Figure 5).

### 3.2. Peak Interfragmentary Compression

A statistically significant difference in PIF was detected (*H* (4) = 35.472, *p* < 0.001). Post hoc analysis revealed no detectable differences in Mean PIF generated between CLS and VPHCS countersunk to 0 mm or −2 mm (0 mm: Mean = 7.512, SD = 6.406, *p* = 0.382; 2 mm: Mean = 17.301, SD = 8.858, *p* = 0.319; CLS: Mean = 12.886, SD = 2.370). The PIF of VPHCSs inserted to depths of 5 mm and 9 mm was greater than that generated by CLSs (5 mm: Mean = 16.086, SD = 6.799, *p* = 0.002; 9 mm: Mean = 34.987, SD = 4.015, *p* < 0.001).

**Figure 6:**
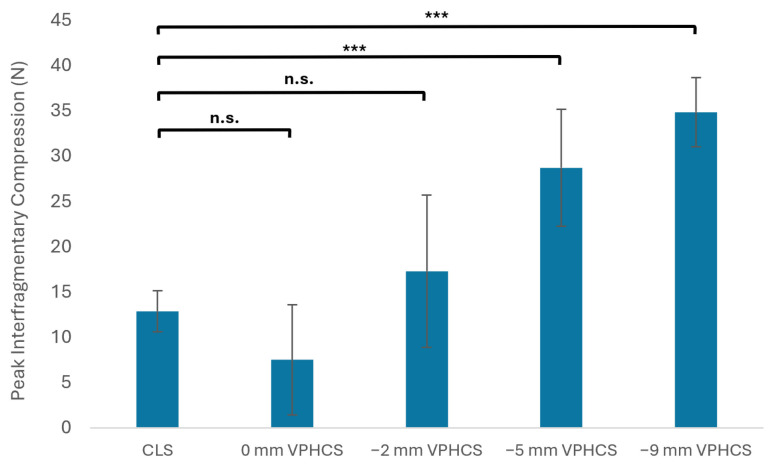
Means and standard deviations for PIF (N) generated by CLSs and VPHCSs countersunk to 0 mm, −2 mm, −5 mm, and −9 mm below the proximal foam surface. For comparison of PIF between groups: *** indicates a significant difference, while n.s. indicates no significance detected between groups.

There was no detectable difference in Mean PIF between VPHCSs countersunk to 0 mm and −2 mm (*p* = 0.061). VPHCSs countersunk to −5 mm produced a significantly greater Mean PIF compared to VPHCSs countersunk to −2 mm (*p* = 0.038). There was no statistical difference in Mean PIF between VPHCSs countersunk to −5 mm and −9 mm (*p* = 0.269) (Figure 6).

### 3.3. Area of Compression

A statistically significant difference in AOC was detected (*H* (4) = 28.736, *p* < 0.001). Post hoc analysis revealed that CLSs produced a statistically greater AOC compared to VPHCSs countersunk to 0 mm (0 mm: Mean = 0.411, SD = 0.322, *p* = 0.045; CLS: Mean = 0.936, SD = 0.291). There was no difference in Mean AOC generated between CLSs and VPHCSs countersunk to −2 mm (2 mm: Mean = 0.925, SD = 0.447, *p* = 0.872). VPHCSs countersunk to −5 mm and −9 mm produced significantly greater Mean AOC compared to CLSs (5 mm: Mean = 1.261, SD = 0.238, *p* = 0.030; 9 mm: Mean = 1.326, SD = 0.159, *p* = 0.006).

**Figure 7:**
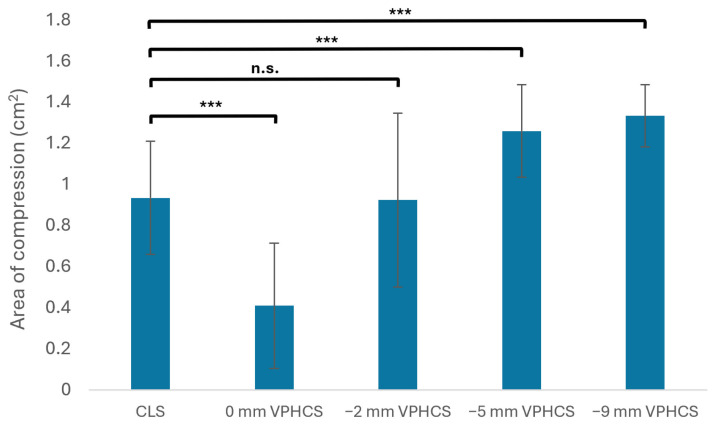
Mean and standard deviation for AOC (cm^2^) generated by CLSs and VPHCSs countersunk to 0 mm, −2 mm, −5 mm, and −9 mm below the proximal foam surface. For comparison of AOC between groups, *** indicates a significant difference, while n.s. indicates no significance detected between groups.

VPHCSs countersunk to 0 mm produced greater AOC compared to VPHCSs countersunk to −2 mm (*p* = 0.031). VPHCSs countersunk to −5 mm produced a significantly greater Mean AOC compared to VPHCSs countersunk to −2 mm (*p* = 0.044). There was no statistical difference detected in Mean AOC between VPHCSs countersunk to −5 mm and −9 mm (*p* = 0.570) (Figure 7).

**Figure 8:**
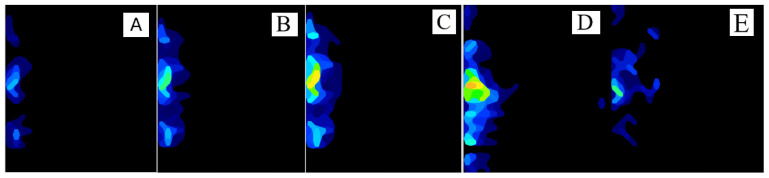
Photographs of compression patterns generated by VPHCSs countersink to 0 mm (**A**), −2 mm (**B**), −5 mm (**C**), −9 mm (**D**), and CLS (**E**), illustrating that for both screws, maximum compressive force is generated immediately adjacent to the screw.

For the VPHCS, the IFC increased in magnitude at each depth, demonstrated by the colour change from blue to green to yellow to orange on compression patterns (Figure 8). The greatest increase in IFC occurred adjacent to the screw; however, forces further away also increased.

### 3.4. Linear Regression

There was a significant, strong positive linear relationship between PIF and VPHCS countersink depth (*F* (1, 38) = 83.450, *p* < 0.001, *r* = 0.829) [42]. There was also a significant, positive linear relationship between AOC and VPHCS countersink depth (*F* (1, 38) = 36.244, *p* < 0.001, *r* = 0.699).

## 4. Discussion

This study compared PIF and AOC generated by fully threaded VPHCSs and CLSs, where the null hypothesis was that there was no significant difference regardless of VPHCS countersink depth. The results of this study showed that CLSs produced significantly greater AOC, but no detectable difference in PIF when compared to VPHCSs countersunk to 0 mm. However, VPHCSs at specific countersink depths produced significantly greater PIF and AOC compared to CLSs; hence, we rejected the null hypothesis. These findings contribute to the growing body of biomechanical investigations evaluating headless compression screws, which have previously been assessed using endpoints such as fixation strength, pushout strength, pullout strength, torsional strength, fastening torque, load/force failure, bending stiffness, and IFC [17,29,31,34,35,37,38,43,44,45,46,47,48].

Whilst a screw is a simple machine, which acts to convert rotation to axial movement, it can possess specific characteristics that generate differing mechanisms of action [1]. CLSs and VPHCSs possess fundamentally different mechanisms of action. A CLS produces IFC due to contact between the head of the screw and the near surface of the bone, preventing translation of the screw through the near fragment [2]. As a result, the near fragment is pulled towards the far fragment, generating compression as the screw continues to be rotated [2].

In contrast, a VPHCS does not abut against the surface of the near fragment. The head of the screw is composed of a conically tapered section, with a smaller thread pitch than that of the body of the screw. As a result, the head can translate through the near fragment as it advances. The body with a larger thread pitch advances further with each rotation relative to the head, which translates to a shorter distance for the same rotation [29]. This results in the proximal fragment being drawn toward the distal fragment, generating IFC. As a result, compression in a VPHCS is governed by the thread pitch differential between the head and tail threads, rather than by a screw head bearing against cortical bone, as seen in conventional CLSs [29].

The VPHCS was inserted according to manufacturer guidelines, using a tapered countersink drill bit, which possibly created a tapered defect wall. This would likely act to distribute forces generated between the head of the screw and the foam evenly along the thread–bone interface in the tapered region of the screw, reducing the propensity for the threads of the “head” to strip. Interestingly, PIF and AOC increased proportionally with VPHCS countersinking depth. This is consistent with Acutrak mini screws, where an increase in both insertional torque and IFC was observed when a screw was countersunk from 0 mm to −2 mm. The present findings align with previous reports, demonstrating that countersinking of VPHCSs increases proportionally with each additional revolution below the surface. Notably, achieving a countersinking depth of at least 2 mm appears to further generate IFC [15,17].

VPHCSs countersunk to −2 mm produced comparable PIF and AOC to CLSs. Previous studies have shown that headless compression screws produce comparable magnitudes of anatomical reduction and stability to CLSs; however, these screws were partially threaded, as opposed to being fully threaded [11,49]. A meta-analysis, which compared the generation of IFC between three types of headless compression screws, found that VPHCS produced statistically greater IFC compared to partially threaded headless compression screws [36]. The findings of the present study are also comparable with those of Cho et al. [44] and Choi et al. [48], who demonstrated comparable fixation strength between fully threaded headless cannulated screws and CLSs in a cadaveric canine humeral condyle and 3D-printed medial malleolar fracture model, respectively. However, as insertion depth was not investigated in both of these studies, further research is warranted to determine its potential impact on fixation strength in cadaveric bone [44,48]. A clinical study is also warranted to see if the observed difference in compression influences the healing response. For a cortical screw, the diameter of a screw may influence compression, as screw fixation is related to the core and thread diameter, the ratio of these diameters, and the thread profile of a screw [50]. For a VPHCS, the screw–bone interface may be influenced by the screw’s core diameter, the outer (thread) diameter, the ratio between these diameters (i.e., the taper), and the thread pitch [33]. Previous studies reported that 3.5 mm VPHCSs produced greater compression forces compared to 2.0 mm CLSs [37]. Similarly, Zindrick et al. [51] demonstrated that the biomechanical strength of screw fixation increases with screw length and diameter. Screws of 3.5 mm diameter were chosen for both CLSs and VPHCSs; however, the present study did not investigate the influence of diameter, nor did it look at specific thread characteristics or profiles. Although the variable core diameter of VPHCSs has been shown to result in significantly greater pullout strength compared with constant core diameter screw designs, its influence on compression generation independent of the screw pitch differential remains unclear [52]. Further research is required to determine the behaviour of VPHCSs of varying screw and core diameters and how it clinically influences compression and the rate of osseous repair.

VPHCSs countersunk to −5 mm and −9 mm produced significantly greater PIF than CLSs. This can be attributed to the VPHCS variable-pitch design: smaller-pitched head threads and larger-pitch point threads. As the VHPCS is progressively countersunk beyond 2–3 mm, it is suggested that the slower-advancing head threads create a cumulative wedge-like compressive effect. The findings of this study support those of previous investigations, that continued advancement of a VPHCS below the cortical surface continues to increase IFC [15,17]. A 9 mm depth was chosen to observe how the screw may impact compression when buried to depths beyond what is recommended in the literature. Loss of compression was not observed at depths of 9 mm in the present study. This could suggest that VPHCSs are more tolerant of over-insertion or “over-tightening”. However, insertion depths beyond 9 mm were not evaluated in this model, as it seemed unlikely that these depths would be encountered in LHC fracture repairs. Additionally, although data quantifying humeral condyle diameters in canines are limited, Cho et al. [44] reported that dogs weighing 6.1–14.4 kg exhibited humeral condyle diameters of 9–14 mm. These findings suggest that insertion of a VPHCS beyond 9 mm may reduce fixation strength, potentially limiting its clinical applicability in dogs within this weight range and with corresponding condylar dimensions. In contrast with traditional CLSs, the head of the screw does not allow continued translation with subsequent rotations, resulting in stripping of the threads in the bone and subsequent loss of IFC and poor fixation. However, bone quality, location, and density might influence these results. Further testing under different densities, quality conditions, and types of material is warranted prior to clinical use.

This study had limitations. In the present study, PU foam of 0.24 g/cm^3^ was selected as a clinically relevant, in vitro model of the bone properties of skeletally immature, small, and toy-breed canines, which account for 70–85% of LHC fracture cases [24,53]. These canines typically exhibit thin cortices and reduced trabecular bone density compared to mature, larger breed dogs such as the Cocker Spaniel, Gordon Setter, and German Short-Haired Pointers, which may be more susceptible to these injuries than other breeds [24,54,55]. PU foam is a useful bone surrogate in biomechanical testing, as it is readily characterised in terms of size, density, and structure, providing a validated material for repeatable testing of mechanical variables. Nevertheless, it is inherently different in composition, structure, and density and does not replicate the anatomy of the humeral condyle [15,56]. Hence, PU foam may possess different mechanical properties than bone in clinical settings. Additionally, bone mineral density may vary significantly with age and sex of canines, which is likely to impact IFC [57,58,59]. Underlying bone pathology, including humeral intercondylar fissures in predisposed large breeds, may also impact compression due to fibrocartilaginous tissue replacement at the sites of fissuring [60]. The density in this study has also been used in human scaphoid experimentation [17]. Results from this test may therefore have relevance to this human scenario as well. Although canine bone has been shown to have fracture stress values similar to human bone, canine and human bones differ in density and composition [61]. Therefore, it is uncertain if the foam accurately represents an in vivo or cadaveric canine humeral trabecular bone. Limitations also arise from repeated PU foam utilisation, particularly with respect to large AOC generated by the VPHCS at countersink depths of −5 mm and −9 mm. Spacing of 10 mm between samples may not have been sufficient in preventing interference from previous tests due to micro-fracture or material fatigue. Surgical technique also posed limitations. A single operator (BC) inserted all screws to standardise insertion. Further research on how varying insertional torque affects PIF magnitude may improve clinical relevance. While torque was used to prevent overtightening and subsequent thread stripping during CLS insertion, torque values were not recorded for the VPHCS, as insertion depth below the foam surface was used as the standardised parameter. However, the authors acknowledge that recording torque values for VPHCSs could have provided additional insight into the relationship between insertion torque, insertional depth, PIF, and AOC; further studies incorporating torque measurements are therefore warranted. Translation of in vitro mechanical studies such as this one must be interpreted with caution. The outcomes measured in this study, i.e., PIF and AOC, were assessed under static conditions at time zero and therefore do not account for potential temporal changes. A longitudinal investigation is required to characterise how the compressive properties of these screws evolve following implantation. In addition, the behaviour of these constructs under different conditions, such as axial compression, bending, and torsion, would be useful in understanding the potential weaknesses of these fixation strategies in the repair of specific fracture configurations. Further work is required to characterise the mechanical behaviour of these screws in in vivo bone tissue. In particular, the influence of physiological loading during weight bearing at different timepoints throughout recovery should be investigated.

Interestingly, as the VPHCS trailing end threads began to engage the distal foam block, separation of foam blocks was observed, but was subsequently followed by a gap reduction with continuous insertion of the screw and generation of compression. This observation could be due to a larger pitch at the point of the screw compared to the head: When the screw point engaged the distal fragment, it advanced further per rotation compared to the screw head. This potentially caused a “push-off” effect where the proximal fragment moved away from the distal fragment until the VPHCS adequately anchored and engaged the distal fragment, whereby compression was generated. Consequently, reduction forceps may prove useful for stabilising the interfragmentary interface and preventing the “push-off” effect during insertion of the VPHCS. The literature has also supported the use of reduction forceps through uniform pressure distribution in CLS fixation and increased generation of IFC in Tibial Plateau Levelling Osteotomy (TPLO) plate fixation [5,10].

## 5. Conclusions

VPHCSs countersunk to either −5 mm and −9 mm produce significantly greater magnitudes of PIF and AOC compared to CLSs in PU with clinical relevance to canine humeral condylar fixation. VPHCSs may serve as an option for fixating articular fractures, such as those involving the humeral condyle. These screws have the potential benefit of avoiding soft tissue impingement or cortical bone resorption, which have been associated with CLSs that rely on compression against the cortical surface; however, further investigation is required to determine recommendations for clinical use and document clinical outcomes.

## Data Availability

The original data presented in the study are openly available in Sydney eScholarship Repository at DOI: 10.25910/z1m3-3281.

## Abbreviations

The following abbreviations are used in this manuscript:

IFC	Interfragmentary compression
CLS	Cortical lag screw
VPHCS	Variable-pitch headless compression screw
PU	Polyurethane
LHC	Lateral humeral condyle
PIF	Peak interfragmentary compression
AOC	Area of compression
